# Clinical Features Related to Severity and Mortality among COVID-19 Patients in a Pre-Vaccine Period in Luanda, Angola

**DOI:** 10.3390/tropicalmed7110338

**Published:** 2022-10-29

**Authors:** Cruz S. Sebastião, Adis Cogle, Alice D’Alva Teixeira, Ana Micolo Cândido, Chissengo Tchoni, Maria João Amorim, N’gueza Loureiro, Paolo Parimbelli, Carlos Penha-Gonçalves, Jocelyne Demengeot, Euclides Sacomboio, Manuela Mendes, Margarete Arrais, Joana Morais, Jocelyne Neto de Vasconcelos, Miguel Brito

**Affiliations:** 1 Centro de Investigação em Saúde de Angola (CISA), Caxito, Angola; 2 Instituto Nacional de Investigação em Saúde (INIS), Luanda, Angola; 3 Instituto de Ciências da Saúde (ICISA), Universidade Agostinho Neto (UAN), Luanda, Angola; 4 Clínica Girassol, Ministério da Saúde, Luanda, Angola; 5 Instituto Gulbenkian de Ciência, 2780-156 Oeiras, Portugal; 6 Católica Biomedical Research Centre, Católica Medical School, Universidade Católica Portuguesa, 1649-023 Lisbon, Portugal; 7 Maternidade Lucrécia Paim, Ministério da Saúde, Luanda, Angola; 8 Hospital Militar Principal, Luanda, Angola; 9 Faculdade de Medicina, Universidade Agostinho Neto (UAN), Luanda, Angola; 10 Health and Technology Research Center, Escola Superior de Tecnologia da Saúde de Lisboa, Instituto Politécnico de Lisboa, 1990-096 Lisbon, Portugal

**Keywords:** SARS-CoV-2, COVID-19, clinical features, Luanda, Angola

## Abstract

Background: Infection due to severe acute respiratory syndrome coronavirus 2 (SARS-CoV-2) is associated with clinical features of diverse severity. Few studies investigated the severity and mortality predictors of coronavirus disease 2019 (COVID-19) in Africa. Herein, we investigated the clinical features of severity and mortality among COVID-19 patients in Luanda, Angola. Methods: This multicenter cohort study involved 101 COVID-19 patients, between December 2020 and April 2021, with clinical and laboratory data collected. Analysis was done using independent-sample *t*-tests and Chi-square tests. The results were deemed significant when *p* < 0.05. Results: The mean age of patients was 51 years (ranging from 18 to 80 years) and 60.4% were male. Fever (46%), cough (47%), gastrointestinal symptoms (26.7%), and asthenia (26.7%), were the most common symptoms. About 64.4% of the patients presented coexistent disorders, including hypertension (42%), diabetes (17%), and chronic renal diseases (6%). About 23% were non-severe, 77% were severe, and 10% died during hospitalization. Variations in the concentration of neutrophil, urea, creatinine, c-reactive protein, sodium, creatine kinase, and chloride were independently associated with severity and/or mortality (*p* < 0.05). Conclusion: Several factors contributed to the severity and mortality among COVID-19 patients in Angola. Further studies related to clinical features should be carried out to help clinical decision-making and follow-up of COVID-19 patients in Angola.

## 1. Introduction

At the end of 2019, the world was confronted with the emergence of cases of pneumonia of unknown etiology initially identified in Wuhan, China [1]. A new coronavirus named Severe Acute Respiratory Syndrome coronavirus 2 (SARS-CoV-2) was identified as being the causative agent of the ongoing outbreak of atypical pneumonia [2,3,4], and the disease was named coronavirus disease 2019 (COVID-19) [5,6]. After identifying the first cases of infection in China, the virus spread rapidly to other geographic locations worldwide acquiring pandemic dynamics and leading to an unprecedented breakdown of healthcare systems with high mortality rates among patients with arterial hypertension, diabetes mellitus, and older age [7,8]. For instance, between December 2019 and May 2022, there have been about 521 million confirmed cases including most of 6.2 million deaths, of which about 99,000 cases and 1900 deaths were reported in Angola [9].

Generally, the main clinical manifestations identified among COVID-19 patients include fever, dry cough, muscle pain, headache, nausea, vomiting, difficulty in breathing, and diarrhea [10,11,12,13]. Furthermore, while those manifestations can be mild or moderate in some patients, they can rapidly evolve into a more severe condition and death in others [14,15,16,17,18,19]. Reportedly, the progression to severe disease has predictable pathology indicators regarding hematological, biochemical, and immunological biomarkers, particularly concerning biological markers of inflammation, impaired liver and kidney function, damage to cardiac tissues and muscles, and hypercoagulation [14,15,16,17,18,19]. Indeed, the pathophysiology of SARS-CoV-2 infection is characterized by aberrant inflammatory responses that affect multiple organs of the cardiac, hepatic, and renal systems leading to unfavorable clinical outcomes [20,21,22].

Studies involving COVID-19 patients around the world have shown that the identification of the laboratory biomarkers of disease progression among COVID-19 patients might be crucial for clinical decision-making with a positive impact on healthcare system costs mainly in low- and middle-income countries. To the best of our knowledge, there are no published studies assessing biomarkers that could be related to the worsening of the disease or unfavorable clinical outcomes among COVID-19 patients in Luanda, the capital city, and the COVID-19 hotspot in Angola. In this study, we identify clinical features related to severity among COVID-19 patients in Angola aiming to contribute to the generation of global knowledge about the clinical effects of SARS-CoV-2 exposure and define effective management strategies for follow-up of COVID-19 patients in Angola.

## 2. Materials and Methods

### 2.1. Study Design and Setting

This was a multicenter cohort study carried out on 101 COVID-19 patients admitted to three hospitals, the Lucrecia Paim Maternity, Hospital Militar Principal, and Clínica Girassol, from December 2020 to April 2021. All health facilities are located in Luanda, Angola. All patients enrolled, have been confirmed as COVID-19 according to the diagnostic criteria established by the WHO, with positive RT-PCR detection in nasal or pharyngeal samples. The study was previously reviewed and approved by the national ethics committee of the Ministry of Health of Angola (approval no. 25/2020). The main inclusion criterion in the study was that participants had to be at least 18 years of age. Moreover, all participants were informed of the study objectives and free verbal consent was obtained from participants before being included in the study.

### 2.2. Sample Collection and Testing

An estimated volume of 10 mL of venous blood was collected from all participants. Of these, 5 mL of blood was placed in tubes containing ethylenediamine tetraacetic acid (EDTA) for the screening of hematological biomarkers (complete blood count or hemogram) using the Automated Hematology Analyzer SYSMEX XT-4000i (Sysmex Europe SE, Norderstedt, Germany). The other 5 mL of blood was placed in tubes with activated clot gel for serum separation and biochemical and/or immunological screening (glucose, urea, creatinine, aspartate transaminase (AST), alanine aminotransferase (ALT), lactate dehydrogenase (LDH), serum creatine kinase (SCK), alkaline phosphatase, albumin, D-Dimer, C-reactive protein (CRP), sodium, potassium, chloride, procalcitonin (PCT), interleukin-6 (IL-6)) using automatic biochemical analyzer Cobas C111 analyzer (Roche), MINI VIDAS (Biomerieux SA, Bagno A Ripoli, Italy) and Cobas E411 (Roche). In addition, we performed the quantification of IgG against SARS-CoV-2 by neutralization assays. The entire process of sample separation, as well as laboratory processing, was carried out in the hematology, biochemistry, and immunology laboratory of Instituto Nacional de Investigação em Saúde (INIS), located in Luanda—Angola. The serological assay for the detection of antibodies that recognize the SARS-CoV-2 Spike protein, by ELISA, was performed using the methodology developed by Florian Krammer [23] at the Instituto Gulbenkian de Ciência, located in Lisbon—Portugal. The baseline laboratory parameters analyzed in these COVID-19 patients were grouped into three major groups, (i) blood routine examination, (ii) serum biochemical index, and (iii) infection-related factors.

### 2.3. Data Sources and Processing

Medical records of all COVID-19 patients were reviewed to collect the sociodemographic (age, gender, and place of residence), clinical information (symptoms, disease severity, comorbidities, and clinical outcome), and laboratory examination results obtained through routine blood tests. The laboratory parameters were analyzed by comparing the average of the values between non-severe and severe patients, as well as between surviving and non-surviving patients. In this study, non-severe patients were those who did not report clinical manifestations but were tested with RT-PCR and included in the study for having an epidemiological link with a confirmed case of SARS-CoV-2 and also for being asymptomatic or pre-symptomatic COVID-19 patients with a high possibility to spreading the infection. On the other hand, patients who revealed any of the symptoms related to SARS-CoV-2 infection were grouped into the category of severe patients. Regarding clinical outcome, we considered surviving patients, all those who were clinically and epidemiologically discharged, while all patients who died during the hospitalization period were grouped as non-survivors.

### 2.4. Statistical Analysis

Statistical analyses were carried out using the SPSS v28 (IBM SPSS Statistics, Armonk, NY, USA). Descriptive data were expressed as frequencies and percentages. Independent-sample *t*-tests were conducted to estimate the differences of continuous data while Chi-square tests were conducted on categorical data. All reported *p*-values are two-tailed with a level of significance of 5%.

## 3. Results

### 3.1. Baseline Characteristics of the Studied Population

As shown in Table 1, the COVID-19 patients from Luanda, Angola, had a mean age of 51 ± 14 years, ranging from 18 to 80 years, most of the patients were male (60.4%, 61/101), and residents of urbanized areas (54.5%, 55/101). A total of 23/101 (23%) patients were non-severe, while 78/101 (77%) were classified as severe. Regarding clinical outcome, a total of 10/101 (10%) patients did not survive during hospitalization and 91/101 (90%) were discharged. The mean age of patients who did not survive was higher compared to those of patients who survived (60 ± 13 years vs. 50 ± 14 years, *p* = 0.045). The most common symptoms at onset were cough (37%), fever (36%), asthenia (27%), gastrointestinal symptoms (27%), dyspnea (19%), headache (15%), osteomyalgia (16%), and fatigue (8%). More than half of patients (64%, 65/101) had some form of the coexisting disorder, with arterial hypertension (42%, 42/101) being the most common coexisting disorder, followed by diabetes mellitus (17%, 17/101) and chronic renal disease (6%, 6/101). Statistically significant differences were observed between the presence of coexisting disorder with the severity of the disease (*p* < 0.001). The top three coexisting disorders in patients who died were arterial hypertension (60%), diabetes mellitus (20%), and chronic kidney disease (20%). Compared to the survivors, the non-survivors were over 40 years old (100%), from urbanized areas (60%), and with a coexisting disorder (90%). Furthermore, another significant difference was observed between the clinical outcome with the presence of chronic kidney disease (*p* = 0.048) or allergic rhinitis (*p* = 0.002). We also explore humoral immune responsiveness by assessing late-stage disease antibodies or Immunoglobulin G (IgG) in approximately 80% of patients (80.2%, 81/101). Immunity assessment results showed that 33% (27/81) had developed an immune response against SARS-CoV-2 and had considerable levels of IgG (mean of 1.67 ± 0.22, ranging from 1.07 to 1.99), while 67% (54/81) had no IgG antibodies. No statistically significant difference was observed between the presence of IgG antibodies and disease severity or clinical outcome. As we expected, the presence of IgG antibodies was more frequently observed among patients with severe disease (37%, 23/78) or in patients who died (44%, 4/10), compared to non-severe patients (21%, 4/23) or patients who survived (32%, 23/91), respectively.

### 3.2. Baseline Laboratory Parameters Related to Disease Severity and Clinical Outcome

Laboratory testing results as well as the average of the laboratory parameters for patients from non-severe vs. severe disease or non-survivors vs. survivors are shown in Table 2. In terms of blood parameters, no significant differences were found between patients classified as non-severe and severe, except for neutrophils (2.40 vs. 5.48, *p* = 0.035). Regarding the biochemical indexes, we observed statistically significant increases in the mean from non-severe patients to severe patients for urea (19.2 vs. 28.1, *p* = 0.017) and CRP (1.57 vs. 7.44, *p* = 0.006), while a significant decrease was observed for sodium (136 vs. 127, *p* = 0.007). A significant increase was observed between survivors and non-survivors for urea (26.5 vs. 29.2, *p* = 0.039), while a significant decrease was observed in creatinine (1.06 vs. 0.50, *p* = 0.025), SCK (230 vs. 136, *p* = 0.039), and chloride (101 vs. 99.7, *p* = 0.026). As we expected, laboratory parameters varied according to gender and age groups. Significant variations for gender were observed with an increase from female to male in AST (31.0 to 55.9, *p* < 0.001), ALT (24.5 to 52.0, *p* < 0.001) and decrease in alkaline phosphatase (105 to 76.5, *p* = 0.029) and chloride (103 to 101, *p* = 0.017). On the other hand, significant variations for the age group were observed with an increase from patients under 40 years to over 40 years in urea (19.7 to 30.9, *p* = 0.003), SCK (155 to 287, *p* = 0.024) and D-Dimer (3.50 to 6.42, *p* = 0.033).

### 3.3. Treatments and Clinical Outcomes among COVID-19 Patients

The therapeutic description used among COVID-19 patients according to gender, age groups, disease severity, and clinical outcomes are described in Table 3. The most used drug groups among the COVID-19 patients analyzed in this study were antibiotics (73%, 74/101), corticosteroids (52%, 51/101), anticoagulants (43%, 43/101), antihypertensives (19%, 19/101), and analgesics (13%, 12/101). Of these therapeutic groups, only antibiotic use was statistically related to clinical outcome, with all non-surviving patients (100%, 10/10) using antibiotics compared to 70% (64/91) of surviving patients exposed to antibiotic therapy. In addition, antibiotics use was also related to disease severity (*p* < 0.001), age group (*p* = 0.025), and gender (*p* = 0.015). Corticosteroid use was related to severity (*p* = 0.001) and age group (*p* = 0.002). Similarly, the use of anticoagulants was related to severity (*p* = 0.001) and age group (*p* = 0.002). Finally, the use of antihypertensive drugs was related to the age group (*p* = 0.029). Curiously, patients treated with antimalarial were part of the group of severe, although the total number is too low to make the result statistically significant.

## 4. Discussion

This extensive, multicenter cohort study was performed among patients with COVID-19 who had a definitive clinical outcome in Angola, a sub-Saharan African country, a continent for which there is a limited number of studies. In the present study, the mean age of all COVID-19 patients was 51 years, which was higher than the mean age reported by Huang et al. (49 years) [16], but lower than that reported by Chen et al. (56 years) [13], and Wang et al. (56 years) [24]. The critically ill patients were mainly older than 40 years old, male, from urbanized regions, and with comorbidities, which resemble findings already reported in Angola by our research group [25]. Furthermore, patients who have the same characteristics related to age and gender have been observed by Zhang et al., in a study conducted in China [12]. As the data are relative to the first wave of the pandemic, it reports data on the first infection of individuals, prior to re-infection or vaccine administrations. Therefore, our data on biological indicators of risk factors associated with worsening and death among COVID-19 patients are free from the confounding effects associated with viral circulating in the population, including prior immunity to the pathogen. Key signs and symptoms as well as the main comorbidities (Table 1) observed in the studied population were in line with many independent reports [12,13,14,15,16]. In contrast with the study carried out by Zhang et al. [12] in which no patient came forward with Rhinitis, our research presented a patient with rhinitis, which was significantly associated with unfavorable clinical outcomes (*p* = 0.002). Currently, we do not have a reasonable explanation of whether allergic conditions such as rhinitis could constitute an independent predictor of mortality amongst COVID-19 patients in Angola. However, additional studies of this possible relationship should be taken into consideration in future studies.

Besides men being those with the most serious disease (Table 1), it was also a group that came forward with a slight decrease in lymphocytes compared with groups of women (0.096), although it is not a statistically significant reduction (Table 2).

Liver damage among COVID-19 patients could affect the C-reactive protein concentrations that were three times higher (5.61 mg/L to 15.2 mg/L) in response to disease severity (Table 2). We observed that the adult age group above 40 years was the group that mostly used antibiotics (Table 3), which could have affected the outcome of these patients, since all patients who died had exposure to antibiotics (*p* = 0.044). All patients who used antimalarial in our study had severe COVID-19 although the total number is too low to make the result statistically significant, which corresponds with previous studies that have seen no benefit and even a trend toward worse clinical outcomes with the use of antimalarial in COVID-19 patients [26,27]. Recently our research team reported a 14% rate of malaria/SARS-CoV-2 coinfection in Luanda [28], which suggests that genetic peculiarities or local diseases such as vector-borne diseases (e.g., malaria, dengue, and chikungunya), might influence the course of the COVID-19 disease representing risk or protective factors for COVID-19 severity and mortality, which deserve further investigation [29]. The biological indicators used to assess responsiveness to infection in these COVID-19 patients were IgG and IL-6. The higher frequency of patients without antibodies IgG is not surprising, as patients were recruited early after disease onset, presumably without having yet developed a humoral response to infection. The increase in IgG antibodies with the severity of the disease is expected and is in accordance with the profile of the immune response to SARS-CoV-2 infection [30,31]. In agreement with our results, Marklund et al. showed that patients with severe COVID-19 seroconvert earlier and develop higher concentrations of SARS-CoV-2-specific IgG compared to patients with non-severe disease, which could improve patient outcomes [30]. Nonetheless, the rate of patients without antibodies (55.6%) who died was higher compared to patients who died despite the presence of antibodies (44.4%), which could indicate that patients who develop IgG antibodies tend to increase their chances of survival. Indeed, a previous study carried out by Corona et al. showed that treatment based on an infusion of IgG enriched with IgM and IgA seems to give a survival advantage in cases of severe infection by SARS-CoV-2 [31].

Our data show a significant difference in sodium concentration in non-severe vs. severe patients (136 mmol/L vs. 127 mmol/L, *p* = 0.007), which is in agreement with a study carried out by Guan et al. where non-severe COVID-19 patients also showed high sodium [31,32]. In our study, patients who died (131 mmol/L) had higher sodium concentrations compared to surviving patients (128 mmol/L) (*p* = 0.403), showing that a high concentration of sodium could be a protective biological factor against an unfavorable clinical outcome. It is also worth mentioning that these results show that during hospitalization, some patients could have developed a state of dehydration which could have led to disturbances in brain function, such as seizures and abnormalities in the level of consciousness. Consistently, loss of consciousness was observed among severe patients and was significantly related to the unfavorable clinical outcome (*p* = 0.002), since the patient with loss of consciousness in this study died during their hospital stay (Table 1).

Generally neglected, variations in sodium concentration could be an indicator of disease severity and have been linked to late hospitalization and significant morbidity [33]. Our results were similar to a study carried out by Albeladi et al. observed low concentrations of sodium in severely COVID-19 patients on admission [34]. A recent study carried out by Chen et al. in China showed that the SARS-CoV-2 infection has a strong association with a decrease in potassium, which was not consistent with the results of this study [35]. Measurement of sodium among severe COVID-19 patients is crucial to avoid complications related to a potassium imbalance, such as dangerous cardiac irregularities [36], once, Moreno-P et al. showed that the reduction of potassium is an indication of disease severity and need for invasive mechanical ventilation [37]. We also observed a significant relationship between the mean concentration of chlorine between surviving and non-surviving patients (*p* = 0.026), indicating that chlorine could be an extremely sensitive biological indicator of SARS-CoV-2 and that reduction could be predictive of bad outcomes. Albeladi et al., also noted that there was a significant decrease in serum chloride values at admission, although during hospitalization the levels increased significantly [34]. In agreement with our results, Petnak et al. showed that serum chloride at hospital discharge in the range of 100–108 mmol/L predicted a favorable clinical outcome [38], which was similar to the mean chlorine concentration of 102 ± 1.03 mmol/L observed among survived patients (Table 2). The reasons for this relationship between chloride concentration and mortality (*p* = 0.026) as well as biological systems with affected biological function due to variation in chlorine concentration among COVID-19 patients have not been explored. Interestingly, there was a decrease in eosinophils with disease severity but an increase in mortality, similar to that seen by Zhang et al. [12], that could also serve as an indicator of infection and mortality.

Previously undertaken studies showed advanced age might be a significant stand-alone predictor of severity and mortality between patients infected with SARS and MERS [39,40,41]. We confirmed that an increase in mean age has been linked to mortality among COVID-19 patients (*p* = 0.045) (Table 1). It is worth noting that all patients who have died were patients aged over 40 years, which represents a group of the largest clinical concerns that require timely intervention from the beginning of the laboratory screening to follow-up during hospitalization. Regarding biological indicators, a significant increase in the concentrations of urea (*p* = 0.003), SCK (*p* = 0.024), and D-Dimer (*p* = 0.033) were observed in the present study among the patients aged over 40 years compared to the younger patients. Nonetheless, we do not know whether these systemic disorders are caused by the fact that patients have COVID-19 or whether there are other genetic, clinical, or behavioral reasons. It is worth mentioning that, during disease progression, the D-dimer significantly increases with the platelets [11]. In this study, we observed increased clotting activity, marked by an increase in D-dimer concentrations by 1.6 times higher in severe COVID-19 patients, 1.8 times higher in patients over 40 years, and a reduction among patients who did not survive (Table 2), which was similar to study carried out by Milbrandt et al. [42] who also observed increased D-dimer in about 90% of hospitalized patients. Our findings support the hypothesis proposed by other authors that SARS-CoV-2 infection activates the coagulation cascade in ways leading to hypercoagulability [11]. On the other hand, our results do not corroborate the association between D-dimer and mortality from COVID-19, reported by Zhou et al. or by Rodelo et al. among COVID-19 patients in Wuhan and Colombia, respectively [14,43].

This study has some caveats. First, the number of participants is low. Second, the patients come from Luanda and might not represent the entire country. Thirdly, due to the limitations in laboratory resources, not all laboratory tests were performed for all patients. Finally, most patients were transferred with high disease severity to health units, and not sampled in this study. Despite these limitations, our study presents the clinical features of COVID-19 patients, explores possible biological indicators related to severity and mortality, allowing an in-depth assessment of the baseline clinical features that might be related to COVID-19 in Angola. Further investigations from a clinical and laboratory point of view must be carried out, to explore and clarify the main laboratory changes that occur during SARS-CoV-2 infection. Furthermore, the possibility of co-infection between viral and bacterial agents and its relationship with severity and clinical outcome should also be investigated in the future. It is also worth mentioning that with the emergence of numerous variants of SARS-CoV-2 with different degrees of infectivity, severity, and mortality, it would be crucial to consider the possibility of exploring the clinical differences and laboratory variations that could occur according to the different variants of SARS-CoV-2.

In conclusion, we identified several biological factors that contributed to the severity and mortality among COVID-19 patients during a period of pre-vaccine in Luanda, Angola. However, further studies related to clinical features, severity, and mortality due to SARS-CoV-2 infection should be carried out to help clinical decision-making and follow-up of COVID-19 patients in Angola.

## Figures and Tables

**Table 1:** Baseline characteristics related to disease severity and clinical outcome among COVID-19 patients in Luanda, Angola.

Baseline Characteristic	N (%)	Disease Severity			Clinical Outcome		
		Non-Severe	Severe	*p*-Value	Survivors	Non-Survivors	*p*-Value
Overall	101 (100%)	23 (22.8)	78 (77.2)		91 (90.1)	10 (9.90)	
Age							
Mean ± SD—yr	51.1 ± 14.2	50.4 ± 13.1	51.3 ± 14.5	0.774	50.2 ± 14.1	59.6 ± 12.5	**0.045**
Distribution—No. (%)							
<20 yr	1 (1.00)	0 (0.0)	1 (1.30)	0.826	1 (1.10)	0 (0.0)	0.161
20–40 yr	24 (23.8)	5 (21.7)	19 (24.4)		24 (26.4)	0 (0.0)	
>40 yr	76 (75.2)	18 (78.3)	58 (74.4)		66 (72.5)	10 (100)	
Gender—No. (%)							
Female	40 (39.6)	11 (47.8)	29 (37.2)	0.359	35 (38.5)	5 (50.0)	0.479
Male	61 (60.4)	12 (52.2)	49 (62.8)		56 (61.5)	5 (50.0)	
Place of residence—No. (%)							
Rural area	46 (45.5)	9 (39.1)	37 (47.4)	0.482	42 (46.2)	4 (40.0)	0.711
Urban area	55 (54.5)	14 (60.9)	41 (52.6)		49 (53.8)	6 (60.0)	
Fever on admission							
Mean (SD)	36.5 ± 0.73	36.3 ± 0.27	36.5 ± 0.81	0.268	36.5 ± 0.69	36.5 ± 1.04	0.955
Distribution of temp.—°C							
<37.5 °C	88 (87.1)	23 (100)	65 (83.3)	0.221	80 (87.9)	8 (80.0)	0.565
37.5–37.9 °C	3 (3.00)	0 (0.0)	3 (3.80)		3 (3.30)	0 (0.0)	
38.0–38.9 °C	9 (8.90)	0 (0.0)	9 (11.5)		7 (7.70)	2 (20.0)	
≥39.0 °C	1 (1.00)	0 (0.0)	1 (1.30)		1 (1.10)	0 (0.0)	
Signs and symptoms—No. (%)	78 (77.2)	0 (0.0)	78 (100)	**<0.001**	68 (74.7)	10 (100)	0.070
Fever	36 (35.6)	0 (0.0)	36 (46.2)	**<0.001**	34 (37.4)	2 (20.0)	0.277
Cough	37 (36.6)	0 (0.0)	37 (47.4)	**<0.001**	32 (35.2)	5 (50.0)	0.355
Headache	15 (14.9)	0 (0.0)	15 (19.2)	**0.023**	14 (15.4)	1 (10.0)	0.649
Fatigue	8 (7.90)	0 (0.0)	8 (10.3)	0.109	7 (7.70)	1 (10.0)	0.798
Asthenia	27 (26.7)	0 (0.0)	27 (34.6)	**<0.001**	23 (25.3)	4 (40.0)	0.318
Dyspnea	19 (18.8)	0 (0.0)	19 (24.4)	**0.009**	16 (17.6)	3 (30.0)	0.340
Osteomyalgia	16 (15.8)	0 (0.0)	16 (20.5)	**0.018**	15 (16.5)	1 (10.0)	0.594
Gastrointestinal symptoms	27 (26.7)	0 (0.0)	27 (34.6)	**<0.001**	24 (26.4)	3 (30.0)	0.806
Apathy	2 (2.00)	0 (0.0)	2 (2.60)	0.438	1 (1.10)	1 (10.0)	0.055
Anosmia	9 (8.90)	0 (0.0)	9 (11.5)	0.088	9 (9.90)	0 (0.0)	0.297
Malaise	20 (19.8)	0 (0.0)	20 (25.6)	**0.007**	16 (17.6)	4 (40.0)	0.091
Hemiplegia	1 (1.00)	0 (0.0)	1 (1.30)	0.585	0 (0.0)	1 (10.0)	**0.002**
Loss of consciousness	1 (1.00)	0 (0.0)	1 (1.30)	0.585	0 (0.0)	1 (10.0)	**0.002**
Coexisting disorder—No. (%)							
No	36 (35.6)	17 (73.9)	19 (24.4)	**<0.001**	35 (38.5)	1 (10.0)	0.074
Yes	65 (64.4)	6 (26.1)	59 (75.6)		56 (61.5)	9 (90.0)	
Disorder distribution—No. (%)							
Chronic pulmonary disease	3 (3.00)	0 (0.0)	3 (3.80)	0.438	3 (3.30)	0 (0.0)	0.636
Arterial hypertension	42 (41.6)	4 (17.4)	38 (48.7)	**0.007**	36 (39.6)	6 (60.0)	0.213
Chronic renal disease	6 (5.90)	0 (0.0)	6 (7.70)	0.170	4 (4.40)	2 (20.0)	**0.048**
Diabetes	17 (16.8)	4 (17.4)	13 (16.7)	0.935	15 (16.5)	2 (20.0)	0.778
Cancer	1 (1.00)	0 (0.0)	1 (1.30)	0.585	1 (1.10)	0 (0.0)	0.739
Immunodeficiency	1 (1.00)	0 (0.0)	1 (1.30)	0.585	1 (1.10)	0 (0.0)	0.739
Hepatitis B infection	1 (1.00)	0 (0.0)	1 (1.30)	0.585	1 (1.10)	0 (0.0)	0.739
Allergic rhinitis	1 (1.00)	0 (0.0)	1 (1.30)	0.585	0 (0.0)	1 (10.0)	**0.002**
IgG							
No	54 (66.7)	15 (78.9)	39 (62.9)	0.194	49 (68.1)	5 (55.6)	0.453
Yes	27 (33.3)	4 (21.1)	23 (37.1)		23 (31.9)	4 (44.4)	

Bold numbers mean that results were statistically significant for independent-sample *t*-tests (*p* < 0.05) and Chi-square tests (*p* < 0.05).

**Table 2:** Baseline laboratory parameters related to disease severity and clinical outcome among COVID-19 patients in Luanda, Angola.

Laboratory Findings	All Patients (101)		Gender			Age Group			Disease Severity			Clinical Outcome		
	N (%)	Mean ± SD	Female (Mean ± SD)	Male (Mean ± SD)	*p*- Value	<40 yr (Mean ± SD)	≥40 yr (Mean ± SD)	*p*-Value	Non-Severe (n = 23)	Severe (n = 78)	*p*-Value	Survivors (n = 91)	Non-Survivors (n = 10)	*p*-Value
**Blood routine examination**														
Erythrocytes, ×10^12^/L	101 (100)	4.62 ± 3.09	4.43 ± 0.95	4.74 ± 1.11	0.140	4.94 ± 1.31	4.52 ± 0.95	0.149	4.77 ± 1.07	4.58 ± 1.06	0.455	4.67 ± 1.05	4.14 ± 1.04	0.131
Hemoglobin, g/dL	101 (100)	12.7 ± 3.09	12.1 ± 2.78	13.1 ±3.25	0.109	13.7 ± 3.81	12.4 ± 2.77	0.113	13.1 ± 3.10	12.6 ± 3.12	0.552	12.8 ± 3.06	11.6 ± 3.37	0.238
Leukocytes, ×10^9^/L	101 (100)	7.44 ± 4.13	6.94 ± 2.97	7.77 ± 4.73	0.282	6.39 ± 3.56	7.79 ± 4.26	0.111	4.57 ± 1.08	7.87 ± 4.66	0.083	6.79 ± 4.16	12.7 ± 4.62	0.079
Neutrophils, ×10^9^/L	98 (97)	4.75 ± 3.40	4.43 ± 2.92	4.97 ± 3.70	0.420	4.34 ± 3.19	4.89 ± 3.47	0.472	2.40 ± 0.17	5.48 ± 4.17	**0.035**	4.40 ± 3.53	10.6 ± 4.60	0.385
Lymphocytes, ×10^9^/L	99 (98)	1.54 ± 0.75	1.69 ± 0.71	1.44 ± 0.77	0.096	1.45 ± 0.70	1.58 ± 0.77	0.440	1.37 ± 0.47	1.61 ± 0.99	0.081	1.62 ± 0.97	1.19 ± 0.18	0.098
Eosinophil, ×10^9^/L	98 (97.0)	0.12 ± 0.27	0.15 ± 0.32	0.10 ± 0.22	0.328	0.10 ± 0.14	0.13 ± 0.30	0.607	0.21 ± 0.49	0.10 ± 0.15	0.078	0.11 ± 0.27	0.21 ± 0.18	0.334
Platelets, ×10^3^/mm^3^	101 (100)	229 ± 122	218 ± 75.5	237 ± 144	0.391	211 ± 83.8	235 ± 132	0.292	154 ± 49.8	270 ± 155	0.212	252 ± 157	255 ± 55.2	0.215
**Serum biochemical index**														
Glucose, mg/dL	96 (95)	126 ± 89.3	124 ± 100	127 ± 82.9	0.896	103 ± 80.4	132 ± 91.1	0.160	117 ± 19.0	148 ± 97.0	0.739	144 ± 92.2	140 ± 95.0	0.512
Urea, mg/dL	93 (92)	28.2 ± 22.3	27.1 ± 29.3	28.9 ± 17.0	0.733	19.7 ± 10.9	30.9 ± 24.3	**0.003**	19.2 ± 1.95	28.1 ± 8.82	**0.017**	26.5 ± 9.20	29.2 ± 0.98	**0.039**
Creatinine, mg/dL	94 (93)	1.45 ± 2.99	2.16 ± 4.85	1.03 ± 0.29	0.176	0.89 ± 3.22	1.63 ± 3.42	0.074	1.03 ± 0.49	0.99 ± 0.38	0.362	1.06 ± 0.36	0.50 ± 0.00	**0.025**
AST, U/L	99 (98)	46.3 ± 38.7	31.0 ± 16.6	55.9 ± 45.1	**<0.001**	49.1 ± 52.6	45.5 ± 33.8	0.755	63.0 ± 32.1	34.6 ± 23.4	0.069	35.4 ± 21.5	70.6 ± 53.0	0.629
ALT, U/L	98 (97)	41.3 ± 45.1	24.5 ± 20.7	52.0 ± 52.7	**<0.001**	46.9 ± 54.1	40.0 ± 42.2	0.554	61.8 ± 18.1	30.7 ± 20.1	0.261	34.4 ± 20.4	43.5 ± 48.7	0.276
LDH, U/L	73 (72)	416 ± 318	379 ± 226	439 ± 364	0.388	317 ± 268	451 ± 329	0.087	291 ± 59.3	380 ± 281	0.091	297 ± 139	991 ± 309	0.114
SCK, U/L	86 (85)	261 ± 326	189 ± 182	303 ± 383	0.066	155 ± 156	287 ± 352	**0.024**	355 ± 168	197 ± 248	0.253	230 ± 252	136 ± 49.3	**0.039**
Alkaline phosphatase, U/L	78 (77)	87.5 ± 50.3	105 ± 62.6	76.5 ± 37.5	**0.029**	117 ± 82.2	79.4 ± 33.7	0.084	79.0 ± 14.1	82.6 ± 52.1	0.394	80.9 ± 50.6	92.8 ± 14.0	0.675
Albumin, g/L	82 (81)	38.3 ± 7.15	37.7 ± 7.79	38.5 ± 6.81	0.645	37.3 ± 9.21	38.5 ± 6.57	0.620	45.8 ± 2.39	35.6 ± 7.69	0.125	37.7 ± 8.27	32.0 ± 0.99	0.052
D-Dimer, g/L	37 (37)	5.47 ± 4.01	5.84 ± 3.51	5.27 ± 4.32	0.671	3.50 ± 3.57	6.42 ± 3.93	**0.033**	2.75 ± 4.01	4.39 ± 3.84	0.274	4.22 ± 3.98	3.45 ± 2.37	0.311
C-reactive protein, mg/L	95 (94)	7.30 ± 15.0	5.56 ± 7.33	8.36 ± 18.1	0.294	4.61 ± 7.04	8.16 ± 16.7	0.153	1.57 ± 1.05	7.44 ± 7.42	**0.006**	5.61 ± 6.54	15.2 ± 8.90	0.099
Sodium, mmol/L	66 (65)	129 ± 8.66	132 ± 10.4	128 ± 7.36	0.070	124 ± 11.5	130 ± 7.86	0.160	136 ± 5.10	127 ± 7.92	**0.007**	128 ± 8.57	131 ± 4.17	0.403
Potassium, mmol/L	60 (59)	8.78 ± 8.38	8.04 ± 9.78	9.12 ± 7.75	0.677	10.1 ± 14.1	8.54 ± 7.13	0.748	3.81 ± 0.54	9.06 ± 9.78	0.082	8.29 ± 9.59	8.07 ± 6.14	0.667
Chloride, mmol/L	65 (64)	102 ± 3.81	103 ± 3.51	101 ± 3.74	**0.017**	101 ± 2.82	102 ± 3.95	0.431	102 ± 1.03	100 ± 3.61	0.061	101 ± 3.55	99.7 ± 1.06	**0.026**
**Infection-related factors**														
PCT, ng/mL	37 (37)	0.62 ± 2.08	1.27 ± 3.78	0.38 ± 0.92	0.251	2.09 ± 4.28	0.22 ± 0.46	0.256	0.09 ± 0.02	1.04 ± 3.01	0.150	0.98 ± 2.93	0.19 ± 0.15	0.900
IL-6, ng/dL	40 (40)	136 ± 314	105 ± 259	151 ± 340	0.477	364 ± 554	60.2 ± 114	0.119	142 ± 231	191 ± 451	0.855	202 ± 441	20.3 ± 19.3	0.524

Abbreviation: AST, aspartate transaminase; ALT, alanine aminotransferase; LDH, lactate dehydrogenase; SCK, serum creatine kinase; PCT, procalcitonin; IL-6, Interleukin 6. Bold numbers mean that results were statistically significant for independent-sample *t*-tests (*p* < 0.05).

**Table 3:** Treatments related to clinical outcomes among COVID-19 patients in Luanda, Angola.

Treatment	Total (n = 101)	Gender			Age Group			Disease Severity			Clinical Outcome		
		Female (n = 40)	Male (n = 61)	*p*-Value	<40 yr (n = 25)	≥40 yr (n = 76)	*p*-Value	Non-Severe (n = 23)	Severe (n = 78)	*p*-Value	Survivors (n = 91)	Non-Survivors (n = 10)	*p*-Value
Antibiotics	74 (73.3)	24 (60.0)	50 (82.0)	**0.015**	14 (56.0)	60 (78.9)	**0.025**	7 (30.4)	67 (85.9)	**<0.001**	64 (70.3)	10 (100)	**0.044**
Corticosteroids	52 (51.5)	14 (35.0)	38 (62.3)	**0.007**	9 (36.0)	43 (56.6)	0.074	5 (21.7)	47 (60.3)	**0.001**	44 (48.4)	8 (80.0)	0.057
Anticoagulant	43 (42.6)	14 (35.0)	29 (47.5)	0.213	4 (16.0)	39 (51.3)	**0.002**	3 (13.0)	40 (51.3)	**0.001**	36 (39.6)	7 (70.0)	0.065
Antihypertensives	19 (18.8)	4 (10.0)	15 (24.6)	0.067	1 (4.00)	18 (23.7)	**0.029**	3 (13.0)	16 (20.5)	0.421	18 (19.8)	1 (10.0)	0.453
Analgesic	12 (11.9)	6 (15.0)	6 (9.80)	0.433	4 (16.0)	8 (10.5)	0.463	1 (4.30)	11 (14.1)	0.204	12 (13.2)	0 (0.0)	0.221
Antiacid	8 (7.90)	3 (7.50)	5 (8.20)	0.899	2 (8.00)	6 (7.90)	0.987	0 (0.0)	8 (10.3)	0.109	6 (6.60)	2 (20.0)	0.136
Antidiabetics	7 (6.90)	1 (2.50)	6 (9.80)	0.156	0 (0.0)	7 (9.20)	0.116	1 (4.30)	6 (7.70)	0.579	7 (7.70)	0 (0.0)	0.363
Antimalarial	5 (5.00)	1 (2.50)	4 (6.60)	0.358	2 (8.00)	3 (3.90)	0.418	0 (0.0)	5 (6.40)	0.213	4 (4.40)	1 (10.0)	0.438
Vitamins	5 (5.00)	2 (5.00)	3 (4.90)	0.985	2 (8.00)	3 (3.90)	0.418	0 (0.0)	5 (6.40)	0.213	5 (5.50)	0 (0.0)	0.447
Antiemetic	3 (3.00)	1 (2.50)	2 (3.30)	0.822	1 (4.00)	2 (2.60)	0.727	0 (0.0)	3 (3.80)	0.340	3 (3.30)	0 (0.0)	0.560

Bold numbers mean that results were statistically significant for Chi-square tests (*p* < 0.05).

## Data Availability

Not applicable.
